# MiR-200c sensitizes Olaparib-resistant ovarian cancer cells by targeting Neuropilin 1

**DOI:** 10.1186/s13046-019-1490-7

**Published:** 2020-01-02

**Authors:** Enrica Vescarelli, Giulia Gerini, Francesca Megiorni, Eleni Anastasiadou, Paola Pontecorvi, Luciana Solito, Claudia De Vitis, Simona Camero, Claudia Marchetti, Rita Mancini, Pierluigi Benedetti Panici, Carlo Dominici, Ferdinando Romano, Antonio Angeloni, Cinzia Marchese, Simona Ceccarelli

**Affiliations:** 1 Department of Experimental Medicine, Sapienza University of Rome, Viale Regina Elena 324, 00161 Rome, Italy; 2 Department of Clinical and Molecular Medicine, Sapienza University of Rome, 00161 Rome, Italy; 3 Department of Maternal and Child and Urological Sciences, Sapienza University of Rome, 00161 Rome, Italy; 4 Department of Public Health and Infectious Diseases, Sapienza University of Rome, 00185 Rome, Italy

**Keywords:** Ovarian cancer, PARP inhibitors, NRP1, miRNAs, Drug resistance, miR-200c

## Abstract

**Background:**

Ovarian cancer (OC) is the most lethal gynecological malignancy and the second leading cause of cancer-related death in women. Treatment with PARP inhibitors (PARPi), such as Olaparib, has been recently introduced for OC patients, but resistance may occur and underlying mechanisms are still poorly understood. The aim of this study is to identify target genes within the tumor cells that might cause resistance to Olaparib. We focused on Neuropilin 1 (NRP1), a transmembrane receptor expressed in OC and correlated with poor survival, which has been also proposed as a key molecule in OC multidrug resistance.

**Methods:**

Using three OC cell lines (UWB, UWB-BRCA and SKOV3) as model systems, we evaluated the biological and molecular effects of Olaparib on OC cell growth, cell cycle, DNA damage and apoptosis/autophagy induction, through MTT and colony forming assays, flow cytometry, immunofluorescence and Western blot analyses. We evaluated NRP1 expression in OC specimens and cell lines by Western blot and qRT-PCR, and used RNA interference to selectively inhibit NRP1. To identify miR-200c as a regulator of NRP1, we used miRNA target prediction algorithms and Pearsons’ correlation analysis in biopsies from OC patients. Then, we used a stable transfection approach to overexpress miR-200c in Olaparib-resistant cells.

**Results:**

We observed that NRP1 is expressed at high levels in resistant cells (SKOV3) and is upmodulated in partially sensitive cells (UWB-BRCA) upon prolonged Olaparib treatment, leading to poor drug response. Our results show that the selective inhibition of NRP1 is able to overcome Olaparib resistance in SKOV3 cells. Moreover, we demonstrated that miR-200c can target NRP1 in OC cells, causing its downmodulation, and that miR-200c overexpression is a valid approach to restore Olaparib sensitivity in OC resistant cells.

**Conclusions:**

These data demonstrate that miR-200c significantly enhanced the anti-cancer efficacy of Olaparib in drug-resistant OC cells. Thus, the combination of Olaparib with miRNA-based therapy may represent a promising treatment for drug resistant OC, and our data may help in designing novel precision medicine trials for optimizing the clinical use of PARPi.

## Background

Ovarian cancer (OC) is the most lethal gynecological malignancy and the second leading cause of cancer-related death in women [1]. About 75% of patients are diagnosed at the late stage of the disease [2, 3], due to the lack of predictive biomarkers. The standard therapeutic protocol, including cisplatin-based combination chemotherapy and cytoreductive surgery, leads to an overall 5-year survival rate of only 15–30% for metastatic OC [4, 5], due to the onset of platinum resistance during the treatment [6]. In recent years, recognition of the role of inherited mutations in the DNA repair genes BRCA1 and BRCA2 in a proportion of OC patients led to the introduction of new therapeutic strategies targeting other DNA repair pathways, using poly-ADP ribose polymerase (PARP) inhibitors, such as Olaparib [7]. PARP inhibitors (PARPi) are able to abrogate PARP functionality, this bringing to the accumulation of single strand breaks (SSBs), which in turn are converted into double strand breaks (DSBs) that cells are not able to repair, causing cancer cell death [8]. PARPi act by blocking the catalytic domain of PARP enzymes, but these agents can also trap PARP proteins on the double-stranded DNA helix, this leading to cytotoxic lesions [9]. This strategy has been approved as treatment option for OC patients bearing BRCA1/2 gene mutation [10]. Moreover, it is now well established that a significant proportion of sporadic ovarian cancers present BRCA-like functional abnormalities (the so-called “BRCAness” syndrome), this opening the possibility of a wider application of treatment regimens specifically designed for familial BRCA-mutated tumors, such as PARPi [11, 12]. However, long-term Olaparib administration might lead to PARPi resistance, which is currently under investigation. To date, the potential mechanisms involved in PARPi resistance are represented by DNA repair restoration [13, 14], PI3K/AKT pathway activation [15] and miRNA dysregulation [16–18], but further investigation is needed to clarify the complexities of pathways underlying PARPi-related clinical resistance.

Neuropilin 1 (NRP1) is a transmembrane glycoprotein belonging to a family of non-tyrosine kinase receptors [19–22], which acts as a receptor for various types of ligands, such as the class 3 semaphorins in neurons, the vascular endothelial growth factor (VEGF) family in endothelial cells, the platelet-derived growth factor (PDGF) in megakaryocytes and the keratinocyte growth factor (KGF) in adipose-derived mesenchymal stem cells [23–25]. NRP1 has been shown to play a critical role in tumorigenesis, cancer invasion and angiogenesis, through the activation of VEGF, PI3K, and AKT pathways [26, 27]. NRP1 protein is highly expressed in different cancer types, such as breast [28], colorectal [22], myeloid leukemia [29], glioma [20], pancreatic [30], and prostate [31] tumors. Some studies also reported an increased NRP1 expression in OC with respect to normal ovarian tissue [32] and to benign ovarian tumors [33]. In OC, NRP1 has been shown to promote unlimited growth through the evasion of contact inhibition [34], and higher NRP1 expression has been correlated with a shorter survival [32, 35], indicating that this protein could be a potential prognostic marker and a molecular target for therapy. Moreover, a comprehensive bioinformatics network analysis demonstrated that NRP1 is involved in multidrug resistance in OC [36].

miRNAs comprise a class of non-coding single-stranded RNAs containing about 21–24 nucleotides, encoded by endogenous genes, which can trigger target mRNA degradation or translation inhibition by targeting its 3′-UTRs [37, 38]. miRNAs are involved in the post-transcriptional regulation of the expression of over 30% of human genes, thus impacting almost every cellular process, and changes in miRNAs expression play a key role in human pathologies, including cancer [39, 40]. In cancer, aberrantly expressed tumor-suppressor or oncogenic miRNAs are involved in tumor progression, metastasis, and drug resistance [41, 42]. So, the modulation of miRNA expression in cancer cells, through inhibiting oncogenic miRNAs or restoring tumor-suppressor miRNAs, could represent a viable approach for improving cancer therapy [43]. Some specific miRNAs, such as miR-148 and miR-124, have been shown to act as upstream suppressors of NRP1 signaling [44, 45]. In cholangiocarcinoma, NRP1 contribution to the growth and metastasis of tumor cells is regulated by miR-320 [46]. In pancreatic cancer, NRP1 is negatively regulated by miR-141, a member of the miR-200 family, and the miR-141/NRP1 axis represents a potentially valuable diagnostic and therapeutic target for this tumor [47]. Almost all the members of miR-200 family (miR-200a, miR-200b, miR-200c and miR-141), have been shown to be upregulated in OC [48]. In particular, the function of miR-200c upregulation in OC [49, 50] is controversial, since it acts as a tumor-promoter by enhancing epithelial-mesenchymal transition, invasiveness, tumor growth and metastasis [51], but on the other hand its overexpression improves the response of OC to various chemotherapeutic agents [52], and its loss is associated with the acquisition of drug resistance [53]. Indeed, an interaction between miR-200 family and NRP1 3’UTR has been previously demonstrated by luciferase reporter experiments and Western blot assays in embryonic stem cells [54], but to date still little is known about the upstream miRNAs regulating NRP1 in OC and the molecular mechanisms by which miRNAs/NRP1 axis modulates drug resistance in this tumor. Therefore, the present study has been designed to investigate if miR-200c could regulate NRP1 in OC, and to assess how miRNA-regulated NRP1 contributes to PARPi resistance.

## Methods

### OC tissue samples

A total of 40 human ovarian specimens was obtained from patients who underwent surgical treatment at the Department of Maternal and Child and Urological Sciences at Sapienza University of Rome between November 2015 and July 2017. Ovarian tumor samples were obtained from n. 28 patients (median age 61 years; range 44–91 years) who underwent radical cytoreductive surgery followed by cisplatin-based chemotherapy (CHT), while normal ovarian samples were obtained from n. 12 patients (median age 61 years; range 53–73 years) with benign ovarian cysts. The 28 patients diagnosed with OC included 26 patients with serous and 2 with mucinous carcinomas. Of the 28 carcinomas, 2 were classified as International Federation of Gynecology and Obstetrics (FIGO) stage II, 21 as stage III, and 5 as stage IV. With regard to the histologic grade, 5 were G1 and 23 were G3. In 16 cases, post-CHT samples were available for study. Patients provided their full consent for the donation of the tissue prior to any surgical procedure. All specimens were stored at − 80 °C until RNA extraction.

### Cell cultures and treatments

The human OC cell lines UWB1.289 (serous, BRCA1-null), UWB1.289 + BRCA1 (serous, BRCA1 restored), and SKOV3 were purchased from the American Type Culture Collection (ATCC-LGC Promochem, Teddington, UK). The UWB cell lines were cultured in a 1:1 mixture of RPMI-1640 (Sigma-Aldrich) and HUMEC (Thermo Fisher Scientific) medium, supplemented with 3% fetal bovine serum (FBS; Invitrogen) and antibiotics. The media for the UWB1.289 + BRCA1 cell line was further supplemented with 200 μg/mL of G-418 solution (Roche Diagnostics, Manheim, Germany) to maintain the expression of the BRCA1 protein. SKOV3 cells were maintained in RPMI (Sigma-Aldrich), supplemented with 10% FBS and antibiotics.

Olaparib (AZD-2281) was purchased from Selleckchem (Suffolk, UK) and used in vitro at concentrations ranging from 1.5 to 10 μM, for the indicated times. Olaparib powder was first dissolved at 10 mM in dimethyl sulfoxide (DMSO; Sigma, St. Louis, MO, USA), diluted to its final concentration with culture medium, and freshly added to the cells every day for the duration of treatment. DMSO alone was used as control in untreated cells at 0.1% (v/v) concentration. Chloroquine (CQ) was purchased from Sigma and used at a final concentration of 10 μM.

### Cell viability assay

Cells were seeded onto 96-well plates at a density of 5 × 10^3^ cells/well, then treated or not with Olaparib for 72 or 144 h at increasing concentrations (1.5, 5 or 10 μM). At the end of time point, cells were incubated with 0.5% MTT (3-(4,5-dimethylthiazol-2-yl)-2,5-diphenyltetrazolium bromide; Sigma, St. Louis, MO, USA) for 4 h at 37 °C. The supernatant was then discarded, the MTT was dissolved with 100 μL of DMSO and absorbance read at OD = 550 nm with an ELISA Microplate Reader (Bio-Rad, Hercules, CA, USA). Cell viability in Olaparib-treated cells was calculated in comparison to control samples (DMSO), arbitrarily set to 100%, having six determinations per assay for each experimental condition.

### Colony formation assay

Cells, previously treated with Olaparib for 144 h, were seeded in 6-well plates in triplicate at a density of 2–4 × 10^3^ cells/well, and incubated at 37 °C for 10–14 days to allow colonies to grow, with medium change every 3 days. Colonies were stained with 0.1% crystal violet for 5 min at room temperature (RT) and photographed. Then, crystal violet was solubilized in 30% acetic acid in water for 15 min at RT, and absorbance was measured using the Biochrom Libra S22 UV/VIS spectrophotometer (Biochrom, Berlin, DE) at a wavelength of 595 nm. 30% acetic acid in water was used as blank control. Colony formation capacity in Olaparib-treated cells was calculated in comparison to control samples (DMSO), arbitrarily set to 1.

### Immunofluorescence analysis

Immunofluorescence was performed as previously described [55]. Cells, treated or not with Olaparib for 144 h, were seeded on coverslips onto 24-well plates at a density of 5 × 10^4^ cells/well, and fixed in 4% paraformaldehyde for 30 min at room temperature, followed by treatment with 0.1 M glycine in PBS for 20 min and with 0.1% Triton X-100 in PBS for additional 5 min to allow permeabilization. Cells were then incubated with anti-phospho-histone H2A.X (Ser139) antibodies (γH2AX; Cell Signalling, Inc. Danvers, MA, USA). After appropriate washing in PBS, primary antibodies were visualized using TexasRed-conjugated goat anti-rabbit IgG (Jackson ImmunoResearch Laboratories, West Grove, PA, USA). Nonspecific fluorescence was determined by omitting primary antibody. Nuclei were visualized using 4′,6-diamidino-2-phenylindole dihydrochloride (DAPI) (Sigma-Aldrich). The single stained and merged images were acquired with a Zeiss ApoTome microscope (40× magnification) using the Axiovision software (Carl Zeiss, Jena, Germany). γH2AX fluorescence intensity was measured using ImageJ software (v. 10.2), evaluating at least six random microscopic fields for each condition.

### Cell cycle and apoptosis analysis by flow cytometry

For cell cycle analysis, cells were treated with 1.5 μM and 5 μM Olaparib for 72 h, then collected and washed twice with phosphate buffered saline (PBS). After fixation in 70% ice-cold ethanol overnight at 4 °C, cell pellets were washed twice with ice-cold PBS and treated with RNase A for 15 min at 37 °C. Propidium iodide (PI) was added to each sample and DNA content was determined by collecting 10,000 events using a BD FACS Calibur Flow Cytometer (BD Biosciences). Data were analyzed using ModFit 3.1 software (BD Biosciences).

Apoptosis was analyzed by using Annexin A5 FITC/7-AAD Kit (Beckman Coulter), following the manufacturer’s instructions. Briefly, cells were treated with Olaparib for 144 h. Approximately 2 × 10^5^ cells were stained with Annexin A5 FITC and 7-Amino-Actinomycin (7-AAD) for 15 min at RT in the dark. Fluorescence intensities of treated samples and controls were collected with a CytoFLEX flow cytometer (Beckman Coulter, Germany). Quadrant analysis was performed using the Kaluza software (Beckman Coulter) to quantify viable cells (7-AAD-negative/Annexin A5-negative), early apoptotic cells (Annexin A5-positive/7-AAD-negative), and late apoptotic cells (Annexin A5-positive/7-AAD-positive). Experiments were performed at least twice. For each point, the sum of early and late apoptotic cells was plotted.

### Western blot analysis

Cells, treated or not with 1.5 μM and 5 μM Olaparib for the indicated times, were lysed in RIPA buffer. Total proteins (50–100 μg) were resolved under reducing conditions by 7–15% SDS-PAGE and transferred to Immobilon-FL membranes (Merck Millipore, Billerica, MA, USA), as previously described [56]. Membranes were blocked in TBS containing 0.1% Tween 20 (TBS-T) and 5% milk for 1 h at 25 °C and then incubated overnight at 4 °C with the following primary antibodies: anti-Neuropilin 1 (A-12) (NRP1) (Santa Cruz Biotechnology, Santa Cruz, CA, USA), anti-phospho-histone H2A.X (Ser139) (γH2AX), anti-Cyclin B1, anti-phospho-AKT (Ser473) (phAKT), anti-AKT, anti-cleaved Caspase-3, anti-cleaved PARP1 (PARP1) (Cell Signalling) and anti-β-Tubulin (Sigma-Aldrich). Membranes were then incubated with the appropriate horseradish peroxidase- (HRP-) conjugated secondary antibody (Santa Cruz Biotechnology) for 1 h at 25 °C. Bound antibody was detected by enhanced chemiluminescence detection reagents (Pierce Biotechnology Inc., Rockford, IL, USA), according to the manufacturer’s instructions. Tubulin served to estimate the protein equal loading. Densitometric analysis was performed using Quantity One Program (Bio-Rad Laboratories S.r.l., Segrate, MI, Italy).

### Quantitative real-time PCR (qRT-PCR)

Cells were harvested and total RNA was extracted with the use of TRIzol reagent (Invitrogen). Quantity and quality of the extracted RNA were assessed by NanoDrop (Thermo Fisher Scientific). For mRNA detection, cDNA was generated with oligo (dT) from 1 μg of RNA using the SuperScript III Reverse Transcriptase Kit (Invitrogen). Quantitative real-time PCR assays (qRT-PCR) were conducted in triplicate on an ABI 7500 Real Time instrument (Applied Biosystems by Life Technologies, Carlsbad, CA, USA) as previously described [57]. Briefly, the abundance of NRP1 was quantified using the appropriate Taq-Man gene expression assay kit (Applied Biosystems). β-actin mRNA was used as endogenous control.

For miRNA detection, 40 ng of RNA were retro-transcribed with a specific primer for miR-200c-3p (Thermo Fisher Scientific). Expression of miR-200c-3p was analyzed by using sequence-specific TaqMan MicroRNA Assays (Applied Biosystems). U6 small nuclear RNA levels were used as internal control.

### siRNA-mediated downregulation of NRP1

The NRP1-specific (siNRP) short interfering RNA, which specifically knock down NRP1 gene expression, as well as negative control siRNA (siNC), which does not lead to the specific degradation of any cellular mRNA, was purchased from Santa Cruz Biotechnology. SKOV3 cells were seeded in 6 well-plate at a density of 0.8 × 10^5^ cells/well and transfected with siRNA at a final concentration of 50 nM using the HiPerfect Transfection Reagent (Qiagen, Valencia, CA, USA) according to the manufacturer’s instructions for long term transfection. The achievement of an efficient knockdown without cytotoxicity at 144 h after the initial transfection was confirmed by performing a time-course experiment. Silenced cells, treated or not with Olaparib (5 μM), were collected and processed for RNA and protein extraction. NRP1 silencing was confirmed by both Western blot and qRT-PCR experiments.

### miRNA conserved target sites prediction in 3′ UTR of NRP1

The RNA22 v2 (https://cm.jefferson.edu/rna22/Interactive/) and the TargetScan (http://www.targetscan.org/vert_72/) predictions were used to identify the putative miRNA target sites in 3′ UTRs of *NRP1* gene. The *NRP1* gene symbol and human species were retrieved from the database. The 3′ UTR of *NRP1* transcript ENST00000374875.1 was selected to analyze the potential binding site of miRNAs.

### Transfection of miR-200c in SKOV3 cell line

Plasmid vector encoding miR-200c and empty pCMV vector were obtained from OriGene Company. Both vectors had Geneticin (G418) resistance as a marker for screening aims. SKOV3 cells were seeded in a 12 well-plate at a density of 0.5 × 10^6^ cells/well and transfected with 1 μg of pCMV-miR-200c plasmid (miR-200c) or the corresponding empty vector (CTRL) using Lipofectamine 3000 (ThermoFisher Scientific), following the manufacturer’s instructions. 48 h post-transfection, cells were resuspended in fresh culture medium supplemented with 0.5 mg/ml G418 and distributed in 96 well-plate. The cells were kept under G418 selection for a couple of weeks in order to obtain G418 resistant clones. One clone from each transfection with pCMV empty vector and pCMV-miR-200c was obtained and used in our studies.

### Statistical analysis

All data reported were verified in at least two different experiments and plotted as means ± standard deviations. The differences between control and experimental groups were analyzed by GraphPad Prism 7, using two-tailed unpaired t test. Pearson’s coefficient correlation was used for correlation assay. *P* values < 0.05 were considered as statistically significant.

## Results

Variable cytotoxic effects of prolonged Olaparib treatment in different OC cell lines are mediated by differential DNA damage repair and activation of apoptosis/autophagy.

We first confirmed the differential effect of Olaparib treatment on OC cell lines depending on BRCA status, by performing a dose- and time-curve evaluation of cell viability through MTT assay in the BRCA1-null UWB1.289 cell line (UWB), the UWB1.289 + BRCA1 cells (UWB-BRCA), in which BRCA1 expression was permanently restored, and the BRCA wild-type SKOV3 cell line. As expected, the sensitivity of the BRCA1-null UWB cells to Olaparib was greater than both its BRCA1 restored counterpart UWB-BRCA and the BRCA wild-type SKOV3 cells (Additional file 1: Figure S1). Olaparib, by inhibiting PARP proteins, rapidly induces DNA damage, which can be measured by γH2AX expression at 24 h, in the three cell lines. In particular, evaluation of γH2AX foci by both immunofluorescence (IF) and Western blot analysis after prolonged Olaparib treatment (144 h) confirmed the persistence of DNA damage only in cells with impaired DNA repair (UWB cells) (Additional file 1: Figure S2). Cell cycle analysis of the three cell lines showed a significant arrest in G2 phase (4n) upon Olaparib treatment, with a corresponding decrease of cell percentage in both G1 (2n) and S phases, particularly evident in UWB and UWB-BRCA cells. Consistent with this observation, cells exposed to Olaparib and, particularly, UWB and UWB-BRCA cells, showed increased expression of Cyclin B1, a G2/M-regulating protein. The distribution of cell cycle fractions in G1, S and G2 phase and the expression of Cyclin B1 are shown in Additional file 1: Figure S3.

**Fig. 1:**
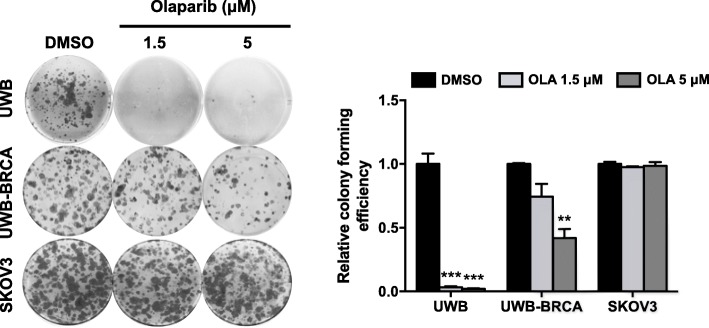
Differential effect of Olaparib on clonogenic ability of OC cell lines. UWB, UWB-BRCA and SKOV3 cells were treated with Olaparib for 144 h, then seeded at low concentration and allowed to grow for 12 days. The effect of Olaparib on cell clonogenicity was determined by colony formation assay. The images are representative pictures of colonies stained with crystal violet. Colony forming efficiency was calculated by crystal violet absorbance. Mean values obtained from two independent experiments, each performed in triplicate, are reported in graph. Error bars represent standard deviations. **, *p* < 0.005, ***, *p* < 0.0005 vs. control (DMSO)

When assessing the effect of prolonged Olaparib treatment (144 h) on colony forming efficiency, we observed that Olaparib significantly inhibited clonogenic ability of BRCA-null UWB cells, while it had no effect on the BRCA wild-type SKOV3 cells, as expected (Fig. 1). However, interestingly, a partial inhibition was achieved at the higher Olaparib dose in UWB-BRCA cells (60% inhibition compared to control samples; Fig. 1).

**Fig. 2:**
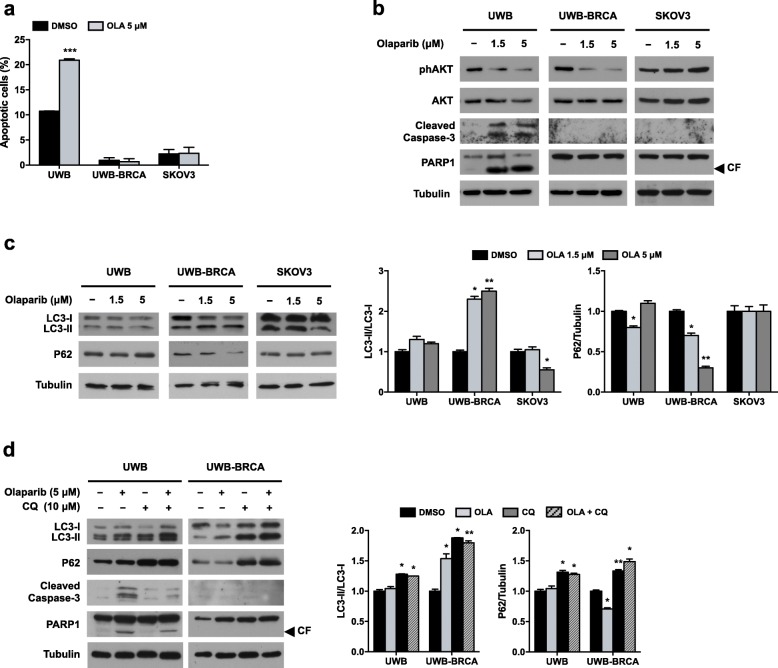
Effects of Olaparib treatment on apoptosis/autophagy induction in OC cell lines. UWB, UWB-BRCA and SKOV3 cells were treated for 144 h with Olaparib. **a**) The percentages of early apoptotic and late apoptotic cells were obtained by flow cytometry quadrant analysis with annexin A5 FITC/7-AAD double staining, and expressed as histograms. **b)** The expression of the apoptosis related proteins phAKT (Ser473), AKT, cleaved Caspase 3 and cleaved PARP1 was determined by Western blot analysis. Tubulin expression was used as internal control. The images are representative of at least two independent experiments. CF, cleaved form of PARP1. **c)** The expression of the autophagy related proteins LC3 and P62 was assessed by Western blot analysis. **d**) UWB and UWB-BRCA cells were treated for 144 h with Olaparib (5 μM), Chloroquine (CQ, 10 μM) or a combination of them, and the expression of LC3 and P62 was assessed. The intensity of the LC3-II and P62 bands was evaluated by densitometric analysis, normalized with LC3-I and Tubulin, respectively, and reported in graph. Error bars represent standard deviations. *, *p* < 0.05, **, *p* < 0.005 vs. control (DMSO)

These data confirmed what previously observed about Olaparib effects [7] but also pointed out a partial efficacy of prolonged Olaparib treatment on UWB-BRCA cells. So, we investigated if the variable response of UWB-BRCA cells upon Olaparib treatment was due to the induction of different intracellular pathways. We assessed the induction of apoptosis by performing flow cytometry assays with Annexin A5 FITC/7-AAD double staining. Treatment of UWB-BRCA and SKOV3 cell lines with Olaparib (5 μM) for 144 h did not significantly increase the percentage of cells undergoing early or late apoptosis when compared to mocked control cells. Indeed, Olaparib treatment induced a consistent increase in the number of apoptotic UWB cells (from about 10% in DMSO to almost 21% in 5 μM Olaparib) (Fig. 2a). So, Annexin A5 FITC/7-AAD double staining confirmed that cytotoxic effects of Olaparib were evident only in UWB cells. Furthermore, Western blot analysis showed that both caspase-3 and PARP1 cleavage/activation were strongly evident only in UWB cells upon Olaparib treatment at both doses (Fig. 2b), in accordance with the data obtained by FACS analysis.

Since PI3K/AKT molecular pathway is a pivotal signal involved in cell survival and apoptosis, we also assessed by Western blot analysis the activation of AKT phosphorylation at Ser473. As reported in Fig. 2b, phosphorylation levels of AKT protein (phAKT) upon treatment with Olaparib were unaffected in SKOV3 cells, and markedly reduced in a dose-dependent way in UWB cells, whilst AKT total levels were unchanged, this confirming the central role of the AKT signal transduction pathway in the Olaparib-mediated cell death. Interestingly, the treatment of UWB-BRCA cells with Olaparib led to a significant downregulation of phAKT expression, without concomitant upregulation of cleaved Caspase 3 and cleaved PARP1 expression.

Since AKT represents a key inhibitor of autophagy, we explored if the reduced phosphorylation state of AKT in UWB-BRCA cells was suggestive of autophagy activation. The classical hallmarks of autophagy, represented by LC3-I to LC3-II conversion and P62 protein degradation, were evaluated through Western blot analysis (Fig. 2c). We observed a significant increase in the LC3-II/LC3-I ratio in UWB-BRCA cells treated with Olaparib 1.5 or 5 μM (2.3 and 2.5 fold, respectively). Autophagy activation in these cells was also confirmed by the degradation of P62 protein (0.7 and 0.3 fold, respectively). Conversely, UWB and SKOV3 cells did not show a significant modulation of both LC3-II/LC3-I ratio and P62 (Fig. 2c). To investigate the involvement of autophagy in PARPi-resistance, we assessed apoptosis induction upon Olaparib treatment in the presence or not of the autophagy inhibitor Chloroquine (CQ). Treatment with CQ induced the accumulation of LC3-II and P62 in both UWB and UWB-BRCA cell lines (Fig. 2d), this reflecting autophagy inhibition. Concurrent treatment with Olaparib and CQ did not induce apoptosis activation in UWB-BRCA cells and significantly decreased the expression of the apoptosis markers cleaved Caspase 3 and cleaved PARP1 in UWB cells (Fig. 2d). These results suggest that in OC cell lines autophagy does not represent a cytoprotective response, but rather contributes to the cytotoxic effect of Olaparib.

### Olaparib treatment modulates NRP1 expression in UWB and UWB-BRCA cells

**Fig. 3:**
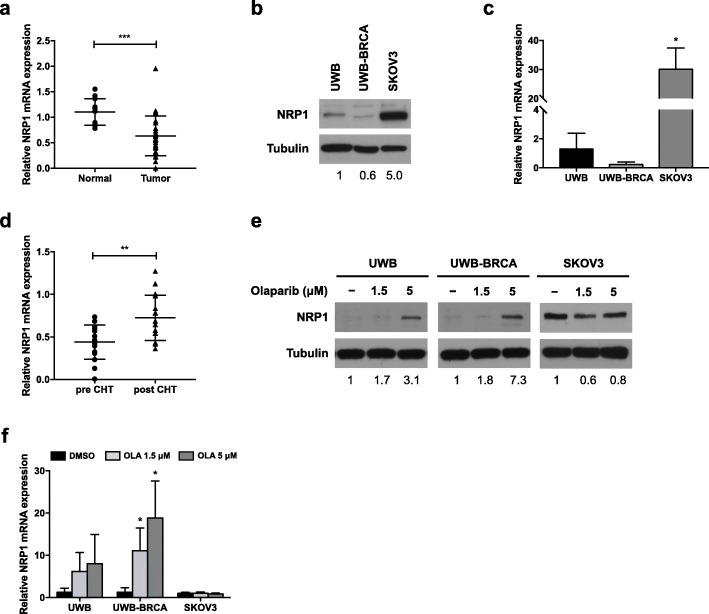
Effect of Olaparib on NRP1 expression in OC. **a**) The expression pattern of NRP1 was assessed by qRT-PCR assays in OC tumor tissues from 28 patients and noncancerous ovarian tissues from 12 healthy donors. ***, *p* < 0.0005. **b, c)** Basal NRP1 protein (**b**) and mRNA (**c**) levels in UWB, UWB-BRCA and SKOV3 cells were assessed by Western blot analysis and qRT-PCR, respectively. **d)** The expression pattern of NRP1 was assessed by qRT-PCR assays in OC tumor biopsies from 16 patients obtained before and after platinum-based chemotherapy (CHT). **, *p* < 0.005. **e, f)** NRP1 protein (**e**) and mRNA (**f**) expression after Olaparib treatment for 144 h were evaluated by Western blot analysis and qRT-PCR, respectively. For Western blot analysis, tubulin expression was used as internal control, and the images are representative of at least three independent experiments. The intensity of the bands was evaluated by densitometric analysis, normalized and reported as relative expression with respect to control (DMSO). For qRT-PCR, each experiment was performed in triplicate and mRNA levels were normalized to GAPDH mRNA expression. Error bars represent standard deviations. *, *p* < 0.05 vs. UWB cells (**c**) or vs control (DMSO) (**f**)

NRP1 is a membrane receptor known to promote tumor growth and drug resistance in several cancers, including OC [19, 30, 34, 36]. Firstly, we performed qRT-PCR to assess the mRNA expression of NRP1 in ovarian cancer specimens. In contrast with previous observations [32], we found that NRP1 expression was significantly lower in OC samples (0.6 fold change with respect to normal ovarian tissue) (Fig. 3a). A low NRP1 protein expression was also confirmed in UWB and UWB-BRCA cells, derived from serous OC, whilst SKOV3 cells, derived from the ascites of an ovarian adenocarcinoma patient, showed a high basal expression of NRP1 protein (5 fold, with respect to UWB cells) (Fig. 3b). This differential expression among the three cell lines was also confirmed at mRNA level by qRT-PCR (Fig. 3c). We then investigated if chemotherapeutic treatment would be able to change NRP1 expression in OC patients. A total of 16 OC samples from the patient cohort were selected, based on the availability of pre- and post-CHT samples, and NRP1 expression was assessed in CHT-naïve and post-CHT OC samples by qRT-PCR. We identified a significantly increased expression of NRP1 mRNA transcript in post-CHT samples (1.7 fold increase with respect to pre-CHT samples) (Fig. 3d). Such correlation between CHT and increase of NRP1 expression supports the hypothesis of a role of NRP1 in drug response and potentially drug resistance. To test this hypothesis in our model, we assessed NRP1 expression in OC cell lines after treatment with Olaparib (1.5 and 5 μM) for 144 h. As shown in Fig. 3e, the treatment induced an upregulation of NRP1 protein in UWB and UWB-BRCA cell lines at both doses (1.7 and 3.1 fold in UWB cells and 1.8 and 7.3 fold in UWB-BRCA cells, respectively). Such upmodulation was confirmed to be significant at mRNA level only in UWB-BRCA cells (Fig. 3f). As for SKOV3 cells, Olaparib treatment did not affect NRP1 protein or mRNA expression (Fig. 3e, f). Our results suggest that Olaparib-mediated upregulation of NRP1 in OC cell lines expressing low basal levels of this protein might represent a drug resistance mechanism.

### NRP1 silencing restores sensitivity to Olaparib treatment in resistant OC cells

**Fig. 4:**
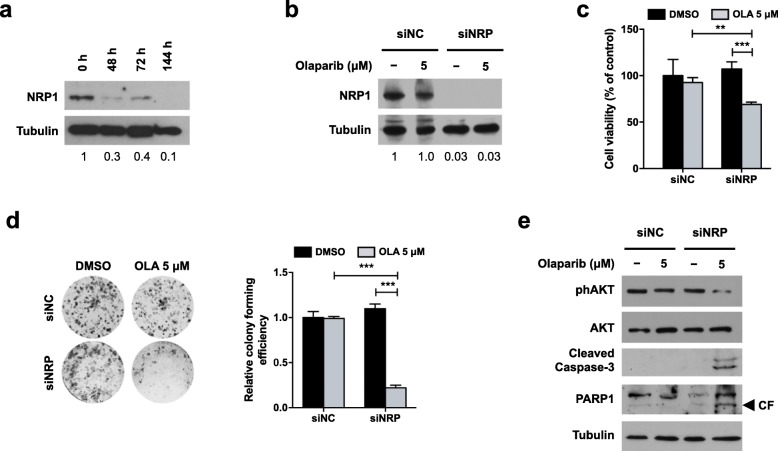
Effect of NRP1 silencing on Olaparib resistance. SKOV3 cells were transfected with NRP1-specific siRNA (siNRP) or nonspecific control siRNA (siNC). **a, b)** NRP1 expression was assessed at 48, 72 or 144 h from transfection (**a**), and after Olaparib treatment at 144 h (**b**) by Western blot analysis. Tubulin expression was used as internal control. The images are representative of at least three independent experiments. **c, d)** The effect of Olaparib on cell viability and clonogenicity of silenced SKOV3 cells was determined by MTT assay and colony formation assay, respectively. Mean values obtained from two independent experiments, each performed in triplicate, are reported in graph. Error bars represent standard deviations. **, *p* < 0.005, ***, *p* < 0.0005 vs. siNC-transfected cells or vs. control (DMSO). **e**) The expression of the apoptosis related proteins phospho-AKT (Ser473), AKT, cleaved caspase 3 and cleaved PARP1 in silenced SKOV3 cells treated or not with Olaparib was assessed by Western blot analysis. Tubulin expression was used as internal control. The images are representative of at least two independent experiments

To confirm the role of NRP1 in Olaparib resistance, we introduced a specific siRNA (siNRP) in the drug-resistant SKOV3 cell line. The efficiency of NRP1 silencing was assessed at 48, 72 and 144 h by Western blot analysis. As reported in Fig. 4a, a significant reduction of NRP1 expression (70%) was achieved as early as 48 h after silencing (0.3 fold with respect to nonspecific control siRNA, siNC), then reached 90% efficiency after 144 h (0.1 fold). NRP1 silencing was also performed on SKOV3 cells untreated or treated with Olaparib (5 μM) for 144 h. Western blot analysis confirmed that Olaparib treatment did not affect NRP1 silencing efficiency (0.03 fold versus siNC in both untreated and treated cells) (Fig. 4b). Then, cells were subjected to both MTT and clonogenic assay, to assess the effect of NRP1 silencing on SKOV3 response to Olaparib. The MTT assay demonstrated that viability of NRP1-silenced cells was reduced by 36% upon Olaparib treatment, whilst siNC-transfected cells treated with Olaparib showed a 7% reduction with respect to DMSO controls (Fig. 4c). As shown in Fig. 4d, Olaparib treatment did not affect clonogenicity of siNC cells, whilst in NRP1-silenced cells it determined a significant reduction in colony formation capacity (0.2 fold with respect to DMSO controls). So, NRP1 silencing was able to restore Olaparib efficacy. Moreover, the role of NRP1 in PARPi resistance is further confirmed by Western blot analysis assessing apoptosis pathway activation (reduction of AKT phosphorylation, cleavage of Caspase 3 and PARP1) in NRP1-silenced SKOV3 cells treated with Olaparib (Fig. 4e). Altogether, these data demonstrate that: i) inhibition of NRP1 restored the sensitivity of drug-resistant cells to Olaparib, ii) NRP1 signaling axis is an important determinant of PARPi tolerance, and iii) modulation of NRP1 expression represents a potential approach to overcome drug resistance in OC.

### NRP1 expression in OC cell lines is regulated by miR-200c

**Fig. 5:**
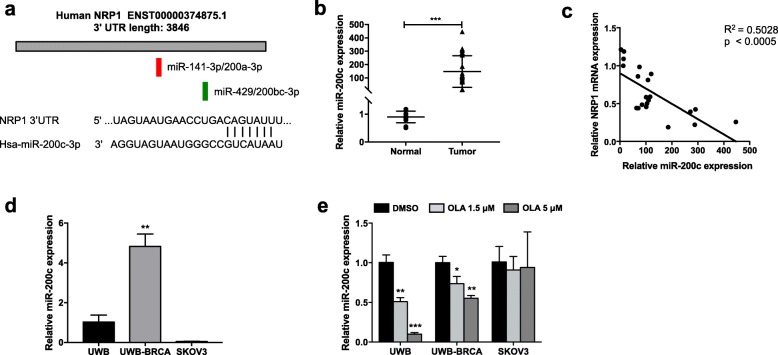
miR-200c-mediated regulation of NRP1 expression in OC cell lines. **a**) Schematic representation of the conserved target sites for miR-141/200a-3p (red) and miR-429/200bc-3p (green) in the 3′ UTR of human NRP1, and Targetscan prediction of miR-200c-3p binding site within the NRP1 3′-UTR. **b)** The expression pattern of miR-200c was assessed by qRT-PCR assays in OC tumor tissues from 28 patients and noncancerous ovarian tissues from 12 healthy donors. ***, *p* < 0.0005. **c)** Correlation analysis between NRP1 and miR-200c expression was performed in OC tumor tissues from 28 patients. **d, e**) UWB, UWB-BRCA and SKOV3 cells were analyzed at basal conditions (**d**) or after Olaparib treatment (**e**), and miR-200c expression was determined by qRT-PCR analysis. Each experiment was performed in triplicate, and miRNA levels were normalized to U6 expression. Error bars represent standard deviations. *, *p* < 0.05, **, *p* < 0.005, ***, *p* < 0.0005 vs. UWB cells (**d**) or vs control (DMSO) (**e**)

As demonstrated above, high expression of NRP1 is correlated with PARPi-resistance of SKOV3 cells. We employed two widely used miRNA target prediction algorithms, RNA22 v2 (https://cm.jefferson.edu/rna22/Interactive/) and TargetScan Human 7.2 (http://www.targetscan.org/vert_72/), to pull potential miRNAs that directly regulate NRP1. In particular, we found two conserved binding sites for the miR-200 family members (one for miR-141-3p/200a-3p and another for miR-429/200bc-3p), which have been previously demonstrated to directly target the 3’UTR of the most prevalent NRP1 transcript [54] (Fig. 5a). Indeed, we focused on miR-200c-3p (from now on referred to as miR-200c), which is predicted to target NRP1 with a percentile score of 68% with 7-nucleotide complementarity (Fig. 5a), since it has been previously identified as an inducer of sensitivity against various anti-cancer agents [52], and its low expression has been implicated in paclitaxel resistance in OC [51]. We observed that the expression level of miR-200c was higher in OC specimens with respect to normal ovarian tissue (Fig. 5b), in accordance to what previously reported [48], and is negatively correlated with that of NRP1 in OC specimens, as shown by correlation analysis (Fig. 5c). Such inverse correlation was also confirmed at protein and mRNA levels in OC cells. In fact, miR-200c basal expression was consistently high in UWB-BRCA cells (5 fold, with respect to UWB cells) and very low in SKOV3 cells (0.05 fold, with respect to UWB cells) (Fig. 5d). The increase of NRP1 expression upon treatment in UWB and UWB-BRCA corresponded to a significant decrease of miR-200c expression in these cells, with no variations in SKOV3 cells, in which Olaparib did not modulate NRP1 levels (Fig. 5e). These data supported the role of miR-200c as a negative regulator of NRP1 in OC.

**Fig. 6:**
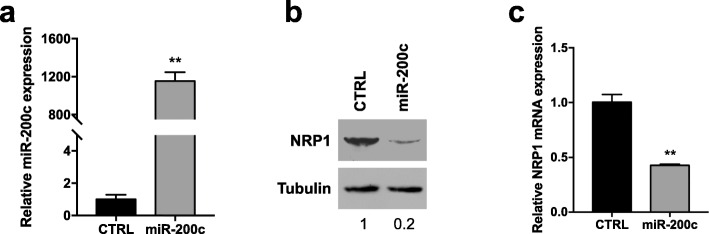
Effect of miR-200c overexpression on NRP1. SKOV3 cells were stably transfected with a plasmid carrying the precursor of miR-200c (miR-200c) and its corresponding vector control (CTRL). miR-200c expression was assessed by qRT-PCR analysis (**a**). NRP1 protein (**b**) and mRNA (**c**) expression were evaluated by Western blot analysis and qRT-PCR, respectively. For Western blot analysis, tubulin expression was used as internal control, and the images are representative of at least three independent experiments. The intensity of the bands was evaluated by densitometric analysis, normalized and reported as relative expression with respect to control (CTRL). For qRT-PCR, each experiment was performed in triplicate. miRNA levels were normalized to U6 expression, while mRNA levels were normalized to GAPDH mRNA expression. Error bars represent standard deviations. **, *p* < 0.005 vs. CTRL

To further validate our hypothesis of NRP1/miR-200c interaction, we investigated the effects of miR-200c overexpression in SKOV3 cells, expressing low levels of miR-200c and high levels of NRP1. First, we confirmed that transfection of miR-200c mimics into SKOV3 cells significantly downmodulated NRP1 expression, as reported in Additional file 1: Figure S4. Cells were then stably transfected with a plasmid carrying the precursor of miR-200c (pCMV-miR-200c) and its corresponding vector control (pCMV), and the expression levels of miR-200c were measured by qRT-PCR. We observed that miR-200c was highly increased in the pCMV-miR-200c group (miR-200c) compared to pCMV empty vector (CTRL) (Fig. 6a), this confirming the miRNA transfection efficiency. Both mRNA and protein expression of NRP1 were assessed in miR-200c-transfected SKOV3 cells by qRT-PCR and Western blot analysis, respectively. Exogenous expression of miR-200c could effectively decrease the levels of NRP1 protein by 80% (Fig. 6b) and of NRP1 mRNA by 60% (Fig. 6c).

### miR-200c sensitizes resistant SKOV3 cells to Olaparib by activating apoptosis

**Fig. 7:**
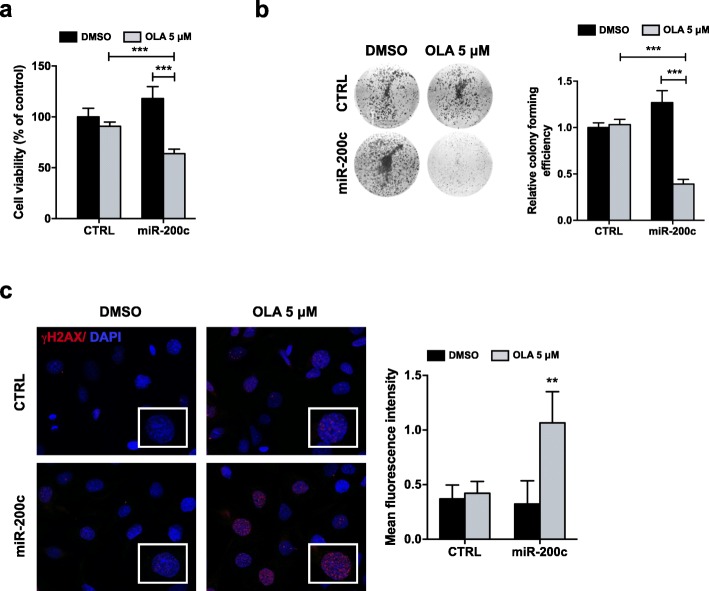
Effect of miR-200c overexpression on SKOV3 response to Olaparib. SKOV3 cells were stably transfected with a plasmid carrying the precursor of miR-200c (miR-200c) and its corresponding vector control (CTRL), then treated for 144 h with Olaparib. **a, b)** Cell viability and colony forming efficiency were determined by MTT assay and clonogenic assay, respectively. Mean values obtained from two independent experiments, each performed in triplicate, are reported in graph. Error bars represent standard deviations. ***, *p* < 0.0005 vs. CTRL cells or vs. control (DMSO). **c)** The presence of γH2AX foci (red) was assessed by immunofluorescence analysis. Nuclei (blue) were visualized with 4′, 6-diamidino-2-phenylindole (DAPI). Images were captured under ApoTome microscope at 40x magnification**.** Quantification of γH2AX foci was determined by measuring red fluorescence intensity with ImageJ software. Mean values obtained from measurements of five microscopic fields randomly taken from three independent experiments are reported in graph. Error bars represent standard deviations. **, *p* < 0.005 vs. control (DMSO)

To confirm the role of miR-200c in Olaparib resistance, SKOV3 cells stably transfected with pCMV-miR-200c (miR-200c) or empty pCMV vector (CTRL) were treated or not with Olaparib (5 μM) for 144 h and used for functional assays. The results of MTT assay revealed that, in miR-200c-transfected SKOV3 cells, Olaparib treatment reduced viability by about 37%, compared to the untreated samples, whilst the reduction of viability after Olaparib treatment was only 9%, compared to DMSO controls, in cells transfected with CTRL vector (Fig. 7a). Similarly, a significant decrease in colony forming efficiency after Olaparib treatment was observed only in miR-200c-transfected cells (0.6 fold with respect to untreated controls) (Fig. 7b). We investigated if miR-200c overexpression would influence SKOV3 ability to repair DNA damage induced by Olaparib. To test this hypothesis, we performed immunofluorescence staining to measure the intensity of γH2AX foci in miR-200c-transfected and CTRL-transfected SKOV3 cells after Olaparib treatment at 144 h. As described above for parental SKOV3 cells, at 144 h γH2AX expression levels in CTRL-transfected cells were similar between Olaparib-treated cells and DMSO controls, while we observed an increase of γH2AX expression after Olaparib treatment in miR-200c-transfected cells (Fig. 7c). Such results suggest that miR-200c overexpression might hinder DNA repair in Olaparib-resistant cells, this leading to a persistent DNA damage and a subsequently greater effectiveness of PARPi.

**Fig. 8:**
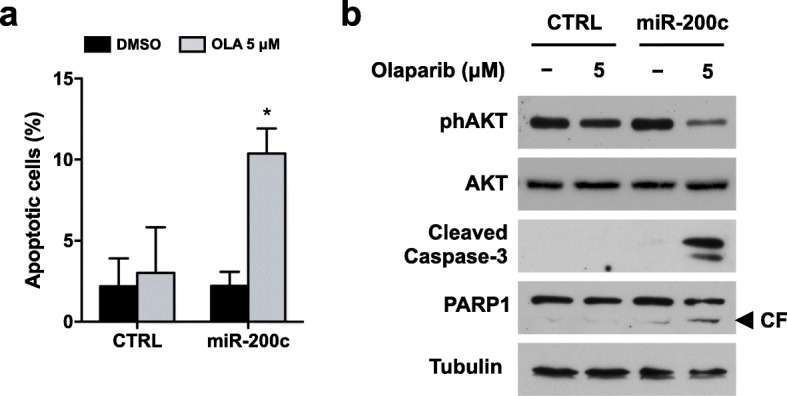
Effect of miR-200c overexpression on Olaparib-induced apoptosis. SKOV3 cells were stably transfected with a plasmid carrying the precursor of miR-200c (miR-200c) and its corresponding vector control (CTRL), then treated for 144 h with Olaparib. **a)** The percentages of early apoptotic and late apoptotic cells were obtained by flow cytometry quadrant analysis with annexin A5 FITC/7-AAD double staining, and expressed as histograms. **b)** The expression of the apoptosis related proteins phAKT (Ser473), AKT, cleaved Caspase 3 and cleaved PARP1 was determined by Western blot analysis. Tubulin expression was used as internal control. The images are representative of at least two independent experiments. CF, cleaved form of PARP1

To confirm such conclusion, we assessed if miR-200c overexpression was able to restore PARPi ability to induce apoptosis. Flow cytometry assays with Annexin A5 FITC/7-AAD double staining demonstrated that Olaparib treatment significantly increased the percentage of apoptotic cells in SKOV3 overexpressing miR-200c (from around 2% in DMSO to approximately 10% in 5 μM Olaparib) and not in CTRL-transfected cells (Fig. 8a). Furthermore, Western blot analysis showed expression of cleaved Caspase 3 and cleaved PARP1 upon Olaparib treatment only in the miR-200c-transfected cells, and not in the CTRL-transfected cells (Fig. 8b). These data strongly suggested that miR-200c regulates apoptosis induction by Olaparib in SKOV3 cells. In agreement with what observed for UWB cells, Olaparib treatment at 144 h did not induce autophagy activation in miR-200c-transfected cells, as reported in Additional file 1: Figure S5.

Altogether, our data suggest that miR-200c overexpression is able to restore SKOV3 susceptibility to Olaparib by targeting NRP1.

## Discussion

OC represents the fifth principal cause of cancer-related death in women [3]. Its high mortality-to-incidence ratio is essentially due to the absence of OC-specific symptoms and the lack of effective screening strategies. Currently, medical options for OC treatment may include a combination of surgery, chemotherapy and radiation. Nevertheless, the general prognosis in OC patients remains poor, with a 5-year survival rate of about 30% [4]. It is known that approximately 10–15% of ovarian cancer patients harbor a germline mutation in genes encoding BRCA1 and BRCA2 proteins, which are involved in the process of homologous recombination (HR) that mediates repair of double stranded DNA breaks [58]. OC patients with BRCA1/2 mutations exhibit impaired ability to repair double-stranded DNA breaks via HR. In this scenario, PARP inhibitors, affecting a second DNA repair pathway, the base excision repair (BER), are able to induce death through a mechanism called synthetic lethality. So, PARPi have been recently approved for treatment of tumors with BRCA1/2 mutations [10]. Nevertheless, the clinical response rates to Olaparib (AZD-2281), a small molecular inhibitor of PARP1, are below 50% among OC patients with confirmed BRCA1/2 inactivation [59, 60], while a subset of patients without germline BRCA mutations might harbor the so-called “BRCAness” phenotype, an impairment in the HR pathway due to other causes (somatic mutations, as well as epigenetic regulations or mutations in other HR pathways) that could be associated with improved response rate and survival after treatment with these drugs [11, 12]. At present, one of the major problems of oncologists is the identification of the most appropriate set of patients that could benefit of the treatment with PARPi. In addition, many OC patients develop local recurrence and distant metastases, often accompanied by resistance to both first-line treatments and PARPi [13, 18]. So, a better understanding of the function of PARP inhibition and the comprehension of drug resistance mechanisms are needed both to predict PARPi clinical efficacy by identifying responsive patients beyond BRCA mutation, and to improve the clinical management of OC patients by introducing novel effective clinical protocols based on combinatorial therapies.

Firstly, we explored the effect of prolonged Olaparib treatment in three OC cell lines and confirmed a differential drug response profile depending on BRCA status. As expected, we observed that treatment with Olaparib is capable of inducing DNA damage and G2/M phase cell-cycle arrest in all the OC cell lines, but such alterations lead to activation of apoptotic pathways only in sensitive UWB cells, maybe due to the persistence of DNA strand breaks, which are restored at 144 h in UWB-BRCA and SKOV3 cells, with subsequent no apoptosis induction following Olaparib treatment. In agreement with our data, lymphoblastoid cells with mono-allelic mutations of BRCA1 showed a persistent DNA damage upon Olaparib treatment, which rendered them hypersensitive to gamma radiation [61]. However, our results pointed out a partial reduction of clonogenic ability induced by Olaparib in UWB-BRCA cells. Interestingly, in these cells we also observed the activation of autophagy upon Olaparib treatment. It is known that autophagy can be upregulated in response to DNA damage, and in the context of cancer it might function as an adaptive cytoprotective response [62], representing a selective advantage for tumor cells by enhancing drug resistance and aggressiveness, or might act as an alternative to apoptosis to eliminate transformed cells [63–65]. In ovarian cancer cells, this alternative role of autophagy has been suggested to depend on the BRCA status [66]. Our data demonstrates that in the partially resistant UWB-BRCA cells, prolonged Olaparib treatment determines autophagy activation, but fails to stimulate apoptosis. Notably, we found that the autophagy inhibitor chloroquine (CQ) did not induce apoptosis activation in Olaparib-treated UWB-BRCA cells, and reduced the ability of Olaparib to induce apoptosis activation in UWB cells, this indicating an essential role of autophagy in Olaparib citotoxicity in OC cells.

It has been previously demonstrated that NRP1 regulation by various miRNAs plays an important role in mediating tumor growth and angiogenesis [44–47]. Moreover, some previous studies reported NRP1 overexpression in OC with respect to normal ovarian tissue [32] and to benign ovarian tumors [33], as well as a correlation between higher NRP1 expression and shorter survival time [32, 35]. However, there is no full consistency among different studies about the correlation of NRP1 expression with OC hystotype or clinicopathological staging. Interestingly, in our work we observed a lower NRP1 expression in OC with respect to normal ovarian tissues in a total cohort of 40 patients, in which we can find a strong prevalence of serous histotype among the 28 OC samples. Our findings on OC tissues are consistent with the observation that NRP1 basal levels are lower in OC cell lines derived from serous OC (UWB and UWB-BRCA cells) with respect to those found in cells from peritoneal adenocarcinoma (SKOV3 cells).

Moreover, a comparative analysis of NRP1 expression between pre- and post-chemotherapy OC samples points out an involvement of NRP1 in OC adaptive response to therapy, this further strengthened by the finding of NRP1 upmodulation in sensitive cells upon prolonged Olaparib treatment. Indeed, we demonstrated that Olaparib could significantly upmodulate NRP1 mRNA and protein only in UWB-BRCA cells, suggesting that in these cells NRP1 expression was sufficient to modulate drug sensitivity, this restricting the effect of Olaparib on cell viability and apoptosis induction. Our data, indicating that NRP1 levels may regulate OC cell lines resistance to PARPi, are in agreement with bioinformatics network analysis suggesting the potential role of NRP1 in drug resistance [36]. Thus, blocking NRP1 expression in OC cells might provide an avenue to increase the sensitivity of drug-resistant cells to Olaparib. Results revealed that downregulation of NRP1 in SKOV3 resistant cells inhibited viability, decreased colony forming potential and induced apoptosis upon Olaparib treatment, indicating that depletion of NRP1 is able to restore sensitivity to PARPi.

Noncoding RNAs, including miRNAs, are endogenous regulatory elements that play a key role in cellular events such as proliferation, differentiation and apoptosis, in both physiological and pathological conditions [39]. In cancer, miRNAs might affect tumor development, progression and drug resistance. Consequently, antagonizing oncogenic miRNAs or restoration of tumor suppressive miRNAs, could represent a reliable tool for improving the cancer therapy [41–43]. However, since each miRNA may regulate several target genes and signaling pathways, miRNA-based treatment requires a careful choice of the potential target.

Increasing evidence show that aberrant expression of miRNAs belonging to the miR-200 family (comprising miR-200a, miR-200b, miR-200c, miR-429 and miR-141) is involved in OC development, as well as in chemoresistance [67–69]. Some of these miRNAs have been shown to interact with NRP1 3′-UTR [54] and to negatively regulate NRP1 signaling [47]. We focused our attention on miR-200c, since it is highly expressed in OC, maybe contributing to epithelial-mesenchymal transition, invasiveness, tumor growth and metastasis [51]. On the other hand, the loss of miR-200c is associated with the acquisition of resistance against various chemotherapeutic agents in different types of cancer, including OC [52]. To date, the function and molecular basis of miR-200c in drug resistance is still undefined. The data obtained about miR-200c expression in our cohort of patients are consistent with previous literature assessing upregulation of miR-200c in OC [48–50], and confirmed an inverse correlation with NRP1 expression.

Our study displayed that overexpression of miR-200c reversed the resistance to Olaparib in OC cells through modulating NRP1 expression. Consistent with our findings, other studies reported a correlation between low expression of miR-200c and paclitaxel resistance in OC [70], and a restoration of paclitaxel sensitivity upon miR-200c overexpression in chemotherapy-resistant cancer cell lines [53, 71, 72] and in a xenograft tumor model [72], this confirming miR-200c as an important control point for the development of chemoresistance in OC.

Our results suggest that tumors with NRP1 downmodulation or miR-200c upmodulation may be more susceptible to PARP inhibition or other strategies based on synthetic lethality. Future studies will determine whether expression levels of NRP1/miR-200c can serve as determinants of therapeutic strategy and of clinical outcome for OC patients.

Indeed, the efficacy in OC cellular models of this novel therapeutic approach, based on the downmodulation of NRP1 through the overexpression of specific miRNA molecules, provides a rationale to translate in vitro experiments in preclinical mouse models. The proposed approach, designed to be administered in combination with PARPi to increase therapeutic efficacy with minimal toxicities, has the potential to develop more effective and less toxic therapeutic protocols for the clinical management of OC patients. Next, we will set up in vivo experiments to test the effects of miR-200c upregulation in overcoming PARPi-related resistance on xenograft mouse models. Future studies will also encompass the role of cancer stem cells (CSCs) in Olaparib resistance mechanisms, by analyzing the efficacy of miR-200c, as monotherapy and in combination with Olaparib, on stemness-related pathways. We think that the development of adjuvant molecular strategies that specifically sensitize CSCs will further contribute to OC eradication.

## Conclusions

In summary, our data describe the PARPi-related response profile of three OC cell lines, clarifying the role of DNA damage, cell cycle arrest and induction of apoptosis/autophagy in PARPi-related resistance, and demonstrate that PARPi sensitivity can be restored by acting on the miR-200c/NRP1 axis. In fact, we showed that miR-200c overexpression increases the response of drug resistant OC cells to Olaparib by targeting NRP1, and that both selective inhibition of NRP1 and stable overexpression of miR-200c might represent a promising approach to improve Olaparib efficacy. Thus, we think that our study will contribute to the design of novel therapeutic strategies for optimizing the clinical use of PARPi in OC patients.

## Additional file

**Additional file 1: Figure S1.** Differential effects of Olaparib treatment on cell viability in OC cell lines. **Figure S2.** Effects of prolonged Olaparib exposure on DNA damage in OC cell lines. **Figure S3.** Induction of G2/M cell cycle arrest by Olaparib in OC cell lines. **Figure S4.** Effect of miR-200c overexpression on NRP1. **Figure S5.** Effect of miR-200c overexpression on autophagy induction

## Data Availability

All data generated during the current study are included within the article.

## Abbreviations

7-AAD: 7-amino-actinomycin D
DMSO: Dimethyl sulfoxide
DSBs: Double strand breaks
miRNA: MicroRNA
NRP1: Neuropilin 1
OC: Ovarian cancer
OLA: Olaparib
PARPi: PARP inhibitors
PI: Propidium iodide
siRNA: Small interfering RNA
SSBs: Single strand breaks
