# Supplementary material for: MiR-200c sensitizes Olaparib-resistant ovarian cancer cells by targeting Neuropilin 1
Source: J Exp Clin Cancer Res. 2020 Jan 2;39:3. doi: 10.1186/s13046-019-1490-7 (PMC6939329; doi:10.1186/s13046-019-1490-7)
Supplement: Supplementary file 1 — Additional file 1: Figure S1. Differential effects of Olaparib treatment on cell viability in OC cell lines. Figure S2. Effects of prolonged Olaparib exposure on DNA damage in OC cell lines. Figure S3. Induction of G2/M cell cycle arrest by Olaparib in OC cell lines. Figure S4. Effect of miR-200c overexpression on NRP1. Figure S5. Effect of miR-200c overexpression on autophagy induction [file 13046_2019_1490_MOESM1_ESM.pdf]

SUPPLEMENTARY MATERIAL FOR

MiR-200c sensitizes Olaparib-resistant ovarian cancer cells by targeting Neuropilin 1

Enrica Vescarelli[1], Giulia Gerini[1], Francesca Megiorni[1], Eleni Anastasiadou[1], Paola Pontecorvi[1], Luciana Solito[1], Claudia De Vitis[2], Simona Camero[3], Claudia Marchetti[3], Rita Mancini[2], Pierluigi Benedetti Panici[3], Carlo Dominici[3], Ferdinando Romano[4], Antonio Angeloni[1], Cinzia Marchese[1], Simona Ceccarelli[1,*]

1. Department of Experimental Medicine, Sapienza University of Rome, 00161, Roma, Italy
2. Department of Clinical and Molecular Medicine, Sapienza University of Rome, 00161, Rome, Italy.
3. Department of Maternal and Child and Urological Sciences, Sapienza University of Rome, 00161, Roma, Italy
4. Department of Public Health and Infectious Diseases, Sapienza University of Rome, 00185, Roma, Italy

SUPPLEMENTARY METHODS

Transfection of oligonucleotides targeting miR-200c

The double-stranded mimics targeting miR-200c, and the negative control (CTRL) oligonucleotides were purchased from Sigma Aldrich. SKOV3 cells were grown to 60-70% confluence, incubated with RNAs at a final concentration of 20 nM by using HiperFect reagent (Qiagen) for 144 h, and then subjected to RNA/protein extraction.

SUPPLEMENTARY FIGURE LEGENDS

Figure S1. Differential effects of Olaparib treatment on cell viability in OC cell lines. UWB, UWB-BRCA and SKOV3 cells were treated for 72 or 144 h with increasing concentration of Olaparib, and cell viability was determined by MTT assay. Mean values of three independent experiments were reported in graph. Error bars represent standard deviations. *, $p < 0.05$, **, $p < 0.005$, ***, $p < 0.0005$ vs. control (DMSO).

Figure S2. Effects of prolonged Olaparib exposure on DNA damage in OC cell lines. a, b) UWB, UWB-BRCA and SKOV3 cells were treated with Olaparib for 24 h (**a**) or 144 h (**b**). The presence of γH2AX foci (red) was assessed by immunofluorescence analysis. Nuclei (blue) were visualized with 4', 6-diamidino-2-phenylindole (DAPI). Images were captured under ApoTome microscope at 40x magnification**.** Quantification of γH2AX foci was determined by measuring red fluorescence intensity with ImageJ software. Mean values obtained from measurements of five microscopic fields randomly taken from three independent experiments are reported in graph. Error bars represent standard deviations. *, $p < 0.05$, **, $p < 0.005$, ***, $p < 0.0005$ vs. control (DMSO). **c)** γH2AX expression after 144 h of Olaparib treatment was determined by Western blot analysis. Tubulin expression was used

as internal control. The images are representative of at least two independent experiments. The intensity of the bands was evaluated by densitometric analysis, normalized and reported as relative expression with respect to control (DMSO).

Figure S3. Induction of G2/M cell cycle arrest by Olaparib in OC cell lines. UWB, UWB-BRCA and SKOV3 cells were treated with Olaparib for 72 h. **a)** Percentages of cells in G1, S and G2 phases were evaluated by flow cytometry. Data are average values of three independent experiments. For G2 increase in Olaparib-treated UWB, UWB-BRCA and SKOV3 cells (both doses), $p < 0.005$ vs. control (DMSO). **b)** The expression of the cell cycle regulatory protein Cyclin B1 was determined by Western blot analysis. Tubulin expression was used as internal control. The images are representative of at least two independent experiments. The intensity of the bands was evaluated by densitometric analysis, normalized and reported as relative expression with respect to control (DMSO).

Figure S4. Effect of miR-200c overexpression on NRP1. SKOV3 cells were transfected with negative control (CTRL) or with miR-200c mimics oligonucleotides for 144 h. miR-200c (**a**) and NRP1 mRNA (**b**) expression were assessed by qRT-PCR analysis. miRNA levels were normalized to U6 expression, while mRNA levels were normalized to GAPDH mRNA expression. NRP1 protein (**c**) was evaluated by Western blot analysis, with tubulin expression as internal control. The intensity of the bands was evaluated by densitometric analysis, normalized and reported as relative expression with respect to control (CTRL). Error bars represent standard deviations. **, $p < 0.005$, ***, $p < 0.0005$ vs. CTRL.

Figure S5. Effect of miR-200c overexpression on autophagy induction. SKOV3 cells were stably transfected with a plasmid carrying the precursor of miR-200c (miR-200c) and its corresponding vector control (CTRL), then treated for 144 h with Olaparib. The expression of

the autophagy related proteins LC3 and P62 was determined by Western blot analysis. Tubulin expression was used as internal control. The images are representative of at least two independent experiments. The intensity of the LC3-II and P62 bands was evaluated by densitometric analysis, normalized with LC3-I and Tubulin, respectively, and reported in graph. Error bars represent standard deviations.

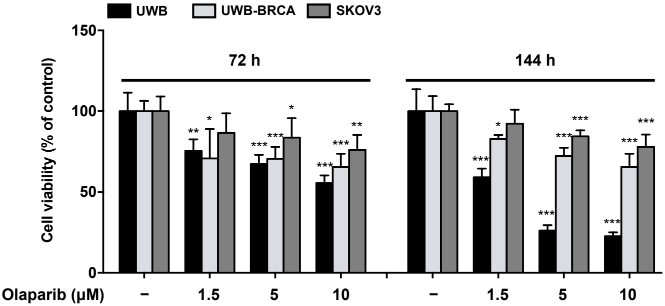
UWB
UWB-BRCA
SKOV3
150
100
50
0
Cell viability (% of control)
72 h
144 h
Olaparib (µM)
–
1.5
5
10
–
1.5
5
10

Fig. S1

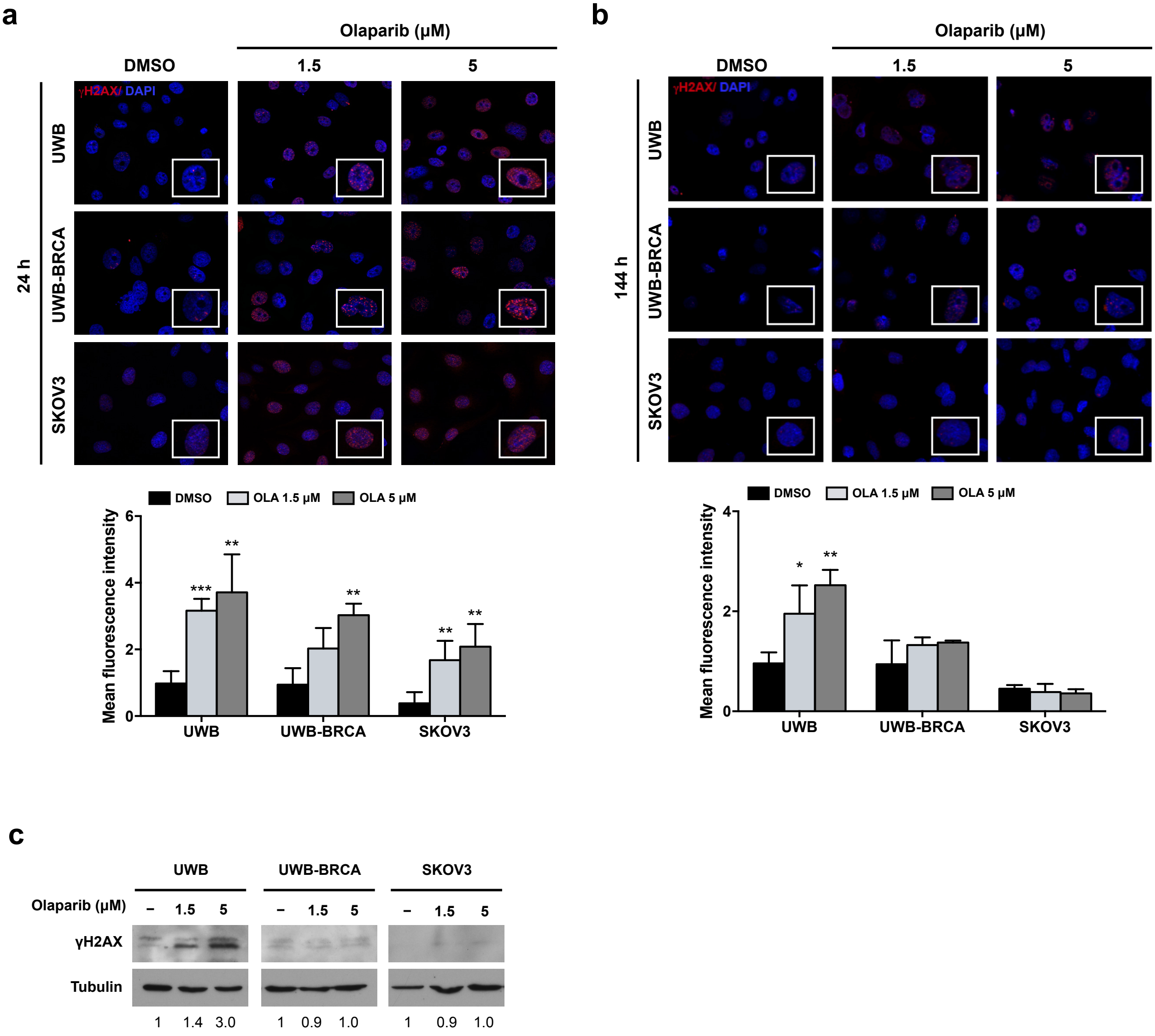
a
Olaparib (μM)
DMSO
1.5
5
γH2AX/ DAPI
24 h
UWB
UWB-BRCA
SKOV3
DMSO
OLA 1.5 μM
OLA 5 μM
Mean fluorescence intensity
0
2
4
6
***
**
**
**
**
UWB
UWB-BRCA
SKOV3
b
Olaparib (μM)
DMSO
1.5
5
γH2AX/ DAPI
144 h
UWB
UWB-BRCA
SKOV3
DMSO
OLA 1.5 μM
OLA 5 μM
Mean fluorescence intensity
0
2
4
*
**
UWB
UWB-BRCA
SKOV3
c
UWB
UWB-BRCA
SKOV3
Olaparib (μM)
– 1.5 5
– 1.5 5
– 1.5 5
γH2AX
Tubulin
1 1.4 3.0
1 0.9 1.0
1 0.9 1.0

Fig. S2

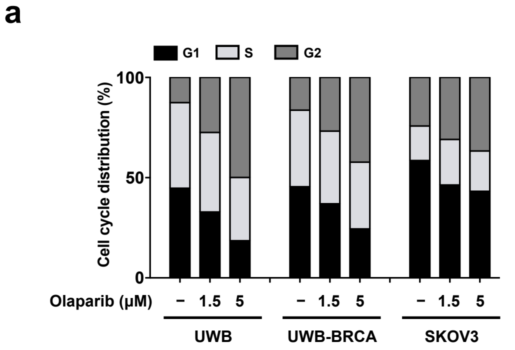
a
G1
S
G2
100
50
0
Cell cycle distribution (%)
Olaparib (μM)
–
1.5
5
UWB
UWB-BRCA
SKOV3

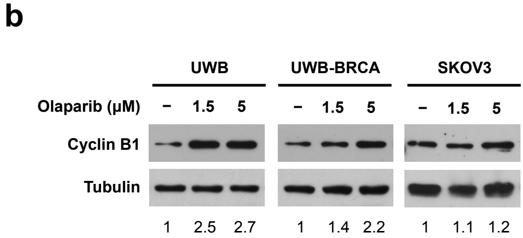
b
UWB
UWB-BRCA
SKOV3
Olaparib (μM)
–
1.5
5
Cyclin B1
Tubulin
1
2.5
2.7
1
1.4
2.2
1
1.1
1.2

Fig. S3

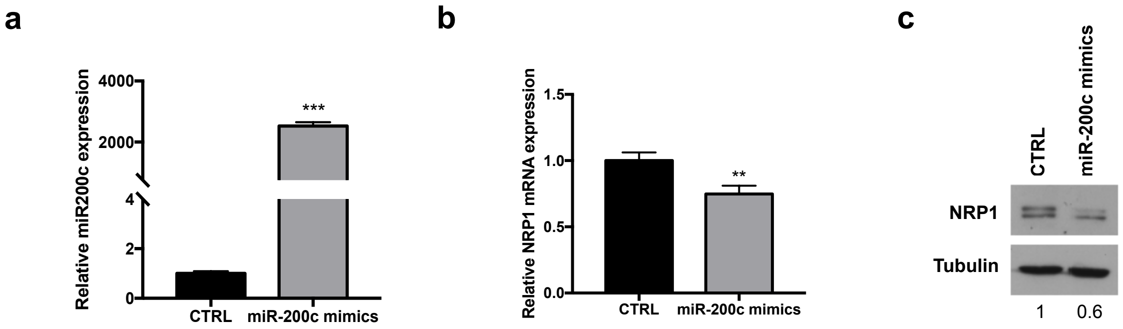
a
Relative miR200c expression
4000
2000
4
2
0
***
CTRL
miR-200c mimics
b
Relative NRP1 mRNA expression
1.5
1.0
0.5
0.0
**
CTRL
miR-200c mimics
c
CTRL
miR-200c mimics
NRP1
Tubulin
1
0.6

Fig. S4

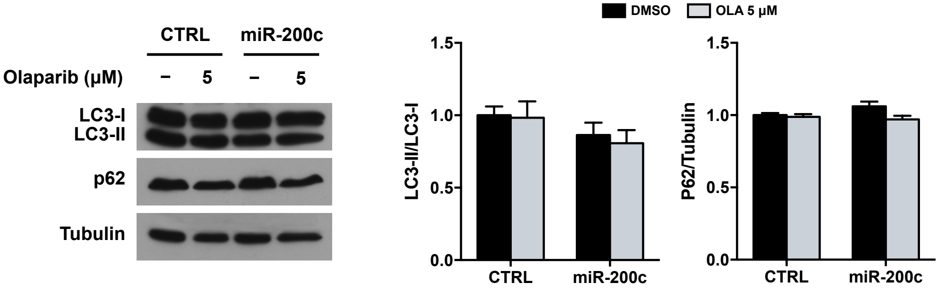
CTRL
miR-200c
Olaparib (μM)
–
5
–
5
LC3-I
LC3-II
p62
Tubulin
DMSO
OLA 5 μM
LC3-II/LC3-I
1.5
1.0
0.5
0.0
CTRL
miR-200c
P62/Tubulin
1.5
1.0
0.5
0.0
CTRL
miR-200c

Fig. S5
